# Supplementary material for: MetaRibo-Seq measures translation in microbiomes
Source: Nat Commun. 2020 Jun 29;11:3268. doi: 10.1038/s41467-020-17081-z (PMC7324362; doi:10.1038/s41467-020-17081-z)
Supplement: Supplementary file 10 — Supplementary Data 7 [file 41467_2020_17081_MOESM10_ESM.zip › File2/Confidence_High_Taxonomy/65120_out.krona.html]

Javascript must be enabled to view this page.

members
magnitude
magnitudeUnassigned
count
unassigned
taxon
rank

65120\_out

3

superkingdom
3
2

32066
phylum
3

203490
class
3

203491
3
order

family
3
1129771

32067
genus
3

712359
3

SRS019071\_contig\_number\_21526SRS057205\_contig\_number\_contig-100\_5359.177024SRS146894\_contig\_number\_15574
species
